# Supplementary material for: MetaRibo-Seq measures translation in microbiomes
Source: Nat Commun. 2020 Jun 29;11:3268. doi: 10.1038/s41467-020-17081-z (PMC7324362; doi:10.1038/s41467-020-17081-z)
Supplement: Supplementary file 10 — Supplementary Data 7 [file 41467_2020_17081_MOESM10_ESM.zip › File2/Confidence_High_Taxonomy/402714_out.krona.html]

Javascript must be enabled to view this page.

members
magnitude
magnitudeUnassigned
count
unassigned
taxon
rank

402714\_out

90

2
90
superkingdom

90
phylum
976

200643
class
90

171549
90
order

90
family
815


SRS053649\_contig\_number\_contig-100\_9106.118916SRS075963\_contig\_number\_contig-100\_3312.3312SRS101433\_contig\_number\_contig-100\_26220.66883SRS148319\_contig\_number\_contig-100\_6261.85230
4
816
90
genus

820

SRS046068\_contig\_number\_857
species
1


SRS018575\_contig\_number\_5816
1
species
376806

species
58

SRS012273\_contig\_number\_1778SRS013098\_contig\_number\_26480SRS013951\_contig\_number\_38419SRS015579\_contig\_number\_15621SRS015794\_contig\_number\_9131SRS015890\_contig\_number\_16819SRS016381\_contig\_number\_2891SRS018984\_contig\_number\_9890SRS022071\_contig\_number\_37171SRS022609\_contig\_number\_48049SRS023715\_contig\_number\_3651SRS024435\_contig\_number\_11991SRS024549\_contig\_number\_11SRS042628\_contig\_number\_32878SRS043001\_contig\_number\_9301SRS043768\_contig\_number\_28192SRS045195\_contig\_number\_1977SRS045713\_contig\_number\_2232SRS045826\_contig\_number\_18831SRS048060\_contig\_number\_2559SRS054590\_contig\_number\_725SRS056519\_contig\_number\_1083SRS058070\_contig\_number\_11383SRS058770\_contig\_number\_3310SRS064232\_contig\_number\_4143SRS064276\_contig\_number\_2430SRS064757\_contig\_number\_22916SRS075341\_contig\_number\_10612SRS077024\_contig\_number\_116SRS077127\_contig\_number\_2050SRS077392\_contig\_number\_40851SRS098655\_contig\_number\_47SRS1041032\_contig\_number\_17163SRS1041038\_contig\_number\_1664SRS1041039\_contig\_number\_21197SRS1041136\_contig\_number\_10623SRS1041137\_contig\_number\_30916SRS1041145\_contig\_number\_38561SRS104636\_contig\_number\_13721SRS1054691\_contig\_number\_13922SRS1055034\_contig\_number\_939SRS1055043\_contig\_number\_9913SRS1055067\_contig\_number\_15917SRS1055099\_contig\_number\_116SRS140513\_contig\_number\_13591SRS143523\_contig\_number\_6412SRS143598\_contig\_number\_19637SRS144135\_contig\_number\_24756SRS147139\_contig\_number\_10082SRS147557\_contig\_number\_34097SRS147653\_contig\_number\_8054SRS148159\_contig\_number\_44144SRS148319\_contig\_number\_3976SRS148511\_contig\_number\_contig-100\_1657.119360SRS149784\_contig\_number\_15790SRS149879\_contig\_number\_17793SRS893230\_contig\_number\_1520SRS971276\_contig\_number\_14710
821

246787
species
1

SRS018133\_contig\_number\_2009

457395
1
species

SRS075021\_contig\_number\_1090

2292939
1
species

SRS019685\_contig\_number\_21308


SRS046717\_contig\_number\_10382SRS098717\_contig\_number\_26
2
species
818

1263042
species
1

SRS050925\_contig\_number\_contig-100\_4367.84858


SRS013476\_contig\_number\_7017SRS014923\_contig\_number\_16196SRS016018\_contig\_number\_88SRS018049\_contig\_number\_76SRS019286\_contig\_number\_19SRS024132\_contig\_number\_1250SRS024140\_contig\_number\_contig-100\_179.179SRS024663\_contig\_number\_1979SRS048981\_contig\_number\_24SRS052078\_contig\_number\_4002SRS078419\_contig\_number\_1483SRS101268\_contig\_number\_364SRS1041112\_contig\_number\_356SRS142712\_contig\_number\_1118SRS148874\_contig\_number\_1553
species
15
357276

2292919

SRS017103\_contig\_number\_41624
species
1

2292935

SRS014613\_contig\_number\_7
1
species

817

SRS013215\_contig\_number\_161SRS013940\_contig\_number\_12593SRS018541\_contig\_number\_1737
3
species
